# Tetherin/BST-2 promotes dendritic cell activation and function during acute retrovirus infection

**DOI:** 10.1038/srep20425

**Published:** 2016-02-05

**Authors:** Sam X. Li, Bradley S. Barrett, Kejun Guo, George Kassiotis, Kim J. Hasenkrug, Ulf Dittmer, Kathrin Gibbert, Mario L. Santiago

**Affiliations:** 1 Department of Medicine, Aurora, University of Colorado Denver, CO 80045, USA; 2 Department of Immunology and Microbiology, University of Colorado Denver, Aurora, CO 80045, USA; 3 The Francis Crick Institute, Mill Hill Laboratory, London, UK; 4 Rocky Mountain Laboratories, National Institutes of Allergy and Infectious Diseases, National Institutes of Health, Hamilton, MT 59840, USA; 5 Institute for Virology, University Hospital in Essen, University of Duisburg-Essen, Essen, Germany

## Abstract

Tetherin/BST-2 is a host restriction factor that inhibits retrovirus release from infected cells *in vitro* by tethering nascent virions to the plasma membrane. However, contradictory data exists on whether Tetherin inhibits acute retrovirus infection *in vivo*. Previously, we reported that Tetherin-mediated inhibition of Friend retrovirus (FV) replication at 2 weeks post-infection correlated with stronger natural killer, CD4+ T and CD8+ T cell responses. Here, we further investigated the role of Tetherin in counteracting retrovirus replication *in vivo*. FV infection levels were similar between wild-type (WT) and Tetherin KO mice at 3 to 7 days post-infection despite removal of a potent restriction factor, Apobec3/Rfv3. However, during this phase of acute infection, Tetherin enhanced myeloid dendritic cell (DC) function. DCs from infected, but not uninfected, WT mice expressed significantly higher MHC class II and the co-stimulatory molecule CD80 compared to Tetherin KO DCs. Tetherin-associated DC activation during acute FV infection correlated with stronger NK cell responses. Furthermore, Tetherin+ DCs from FV-infected mice more strongly stimulated FV-specific CD4+ T cells *ex vivo* compared to Tetherin KO DCs. The results link the antiretroviral and immunomodulatory activity of Tetherin *in vivo* to improved DC activation and MHC class II antigen presentation.

Restriction factors are host-encoded type I interferon-stimulated genes (ISG) that directly inhibit virus replication^1^. Their importance was emphasized by the existence of viral antagonists, as exemplified by pandemic HIV-1 strains encoding Vpu to counteract the restriction factor Tetherin (also known as BST-2, CD317, HM1.24, and PDCA-1)^2^,^3^. Tetherin is a type II membrane protein that can inhibit retrovirus release and spread *in vitro*. It consists of an N-terminal cytoplasmic tail, a transmembrane region, an extracellular domain containing cysteines necessary for dimerization, and a C-terminal glycophosphatidyl inositol anchor^4^. Tetherin’s unique molecular structure allows it to physically ‘tether’ the viral membrane to the host cell membrane, thereby retaining the virus on the cell surface. While Tetherin’s antiviral function was based on this tethering mechanism, the impact of virus particle retention on retroviral replication is controversial. While some *in vitro* studies reported that Tetherin restricted retroviruses^2^,^3^,^5^, others showed that Tetherin enhanced cell-to-cell spread^6^,^7^,^8^,^9^. Conflicting conclusions regarding Tetherin’s impact on retrovirus replication *in vitro* prompted the need to study Tetherin biology *in vivo.* Murine retrovirus infection models involving Friend retrovirus (FV), ‘murine AIDS’ (LP-BM5), mouse mammary tumor virus (MMTV) and moloney murine leukemia virus (Mo-MuLV) were particularly attractive as these models allowed for performing direct causation studies using Tetherin knockout (KO) mice.

Previous studies comparing retrovirus infection levels in wild-type (WT) versus Tetherin KO mice revealed contradictory results. Two studies found that WT and Tetherin KO mice had no significant difference in acute LP-BM5 and/or Mo-MuLV replication^10^,^11^, while another study found that Tetherin KO mice had higher acute MMTV replication levels^12^. Interestingly, Liberatore and Bieniasz found that even though WT and Tetherin KO mice had similar acute LP-BM5 replication levels, Tetherin KO mice had higher infection levels during later time points, when adaptive immune responses operate^10^,^13^. These data raised the possibility that Tetherin may be modulating the adaptive immune response. The notion that an innate restriction factor can modulate adaptive immunity is not unprecedented, as the restriction factor mouse Apobec3 (or mA3) has been shown to augment FV-specific neutralizing antibody responses^14^,^15^.

We recently provided evidence that Tetherin could promote innate and adaptive cell-mediated immune responses against FV infection^16^. FV is a complex of a replication-competent but non-pathogenic helper Friend MuLV (F-MuLV), and a replication-defective but pathogenic spleen focus forming virus (SFFV). FV infects adult immunocompetent mice and causes splenomegaly and erythroleukemia^17^. Classical restriction genes such as Fv2 and mA3/Rfv3 strongly influence the susceptibility of mice to FV disease^17^. C57BL/6 (B6) mice encode resistant forms of Fv2 and mA3/Rfv3, which significantly inhibit splenomegaly induction^18^ and promote neutralizing antibody responses^14^,^15^, respectively. However, B6 mice remain susceptible to infection and erythroleukemia especially at high FV inoculum dose, older age^19^ and compromised CD8+ T cell responses^20^. In addition to CD8+ T cell responses, NK cell and CD4+ T cell responses are also required for effective control of FV infection in B6 mice^21^,^22^,^23^,^24^,^25^,^26^.

During peak T cell responses to FV, Tetherin KO mice had weaker IFNγ expression in NK cells, CD4+ T cells, and CD8+ T cells, and weaker cytotoxic responses in NK and CD8+ T cells^16^. In addition, Tetherin KO mice had reduced numbers of virus-specific CD8+ T cells. These cell-mediated immune responses correlated with lower plasma viral loads and cellular infection levels. These results demonstrated a role for Tetherin in promoting the cell-mediated immune response to retroviral infection. However, it remained unclear whether Tetherin had a direct effect on acute FV replication. Higher FV replication in Tetherin KO versus WT mice during early stages of the infection may result in weaker cell-mediated immune responses in Tetherin KO mice due to higher FV-induced immune dysfunction.

Dendritic cells (DCs) play key roles in priming both NK and T cell responses^27^,^28^ and are susceptible to FV infection *in vivo*^29^. Thus, an effect of Tetherin on DC function may govern its ability to stimulate cell-mediated immune responses. In the current study, we explored Tetherin’s role in early retroviral infection, when DCs were at the crucial stage of priming cell-mediated immune responses. Utilizing B6 WT and Tetherin KO mice, we show that Tetherin had no effect on early retrovirus replication, but promoted DC function. Our results highlight Tetherin-mediated DC activation as a critical mechanism for how Tetherin influenced retrovirus cell-mediated immune responses that subsequently inhibited retrovirus replication *in vivo*.

## Results

### Tetherin does not influence acute FV infection levels

**Figure 1:**
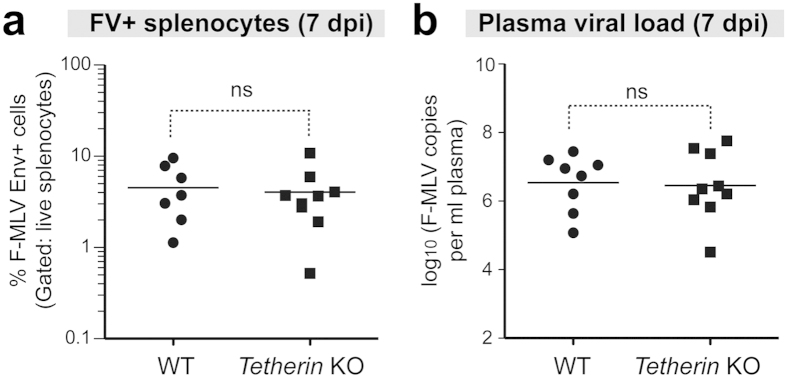
Tetherin does not inhibit acute FV infection. WT and Tetherin KO mice were infected with 10^4^ SFFU of FV. At 7 dpi, splenocytes were stained with the F-MuLV env-specific mAb 720 for flow cytometry. (**a**) Comparison of % F-MuLV env+ splenocytes from WT and Tetherin KO mice. (**b**) Plasma viral load comparison between WT and Tetherin KO mice. Plasma samples at 7 dpi were evaluated for F-MuLV RNA copies by qPCR. Absolute viral copies were determined using an F-MuLV RNA standard. Log-transformed values are shown. In both panels, lines represent means, with each dot representing data from an individual mouse. Statistical analyses were performed using a 2-tailed Student’s *t* test. Data for each group were combined from 2 independent experiments. *p < 0.05; ns, not significant (*p* > 0.05).

Innate restriction factors are expected to counteract virus infection prior to the onset of adaptive immune responses. Tetherin is widely considered as an innate restriction factor and its expression is significantly induced in bone marrow and spleen 24 h after FV infection^16^. We therefore determined if Tetherin could inhibit acute FV infection levels *in vivo*. B6 WT and Tetherin KO mice were infected with FV and analyzed at 7 days post infection (dpi). Splenocytes from these mice were stained for surface expression levels of F-MuLV env and analyzed by flow cytometry. F-MuLV env+ cells were quantified using gates set with a stained uninfected sample as <1% as previously described^30^. The percentage of F-MuLV env+ splenocytes was not significantly different between WT and Tetherin KO mice (Fig. 1a). Viral RNA copies in plasma were also found to be similar between WT and Tetherin KO mice (Fig. 1b). Thus, Tetherin did not significantly influence acute FV infection levels.

### **Tetherin does not activate NF-**κ**B in mouse NIH3T3 cells**

We previously showed that Tetherin inhibited FV replication at 14 dpi^16^. Since Tetherin had no effect on FV infection levels at 7 dpi (Fig. 1), the effect of Tetherin at a later time point may not be due to its direct restriction properties. Notably, Tetherin-mediated inhibition of virus replication at 14 dpi correlated with stronger NK cell and virus-specific CD8+ T cell responses^16^. The underlying mechanism for how Tetherin influenced cell-mediated immunity remains unclear. Recently, human Tetherin was found to activate NF-κB, which controls many immunity-related genes^31^,^32^,^33^,^34^. Mouse Tetherin could not activate NF-κB, but the assays were performed in human cells^31^. We therefore tested if mouse Tetherin could activate NF-κB in murine NIH3T3 cells. Similar to findings in human 293T cells, human Tetherin induced NF-κB in murine NIH3T3 cells, but mouse Tetherin did not (Supplementary Fig. 1 online).

### Tetherin does not alter DC phenotypes in the absence of infection

**Figure 2:**
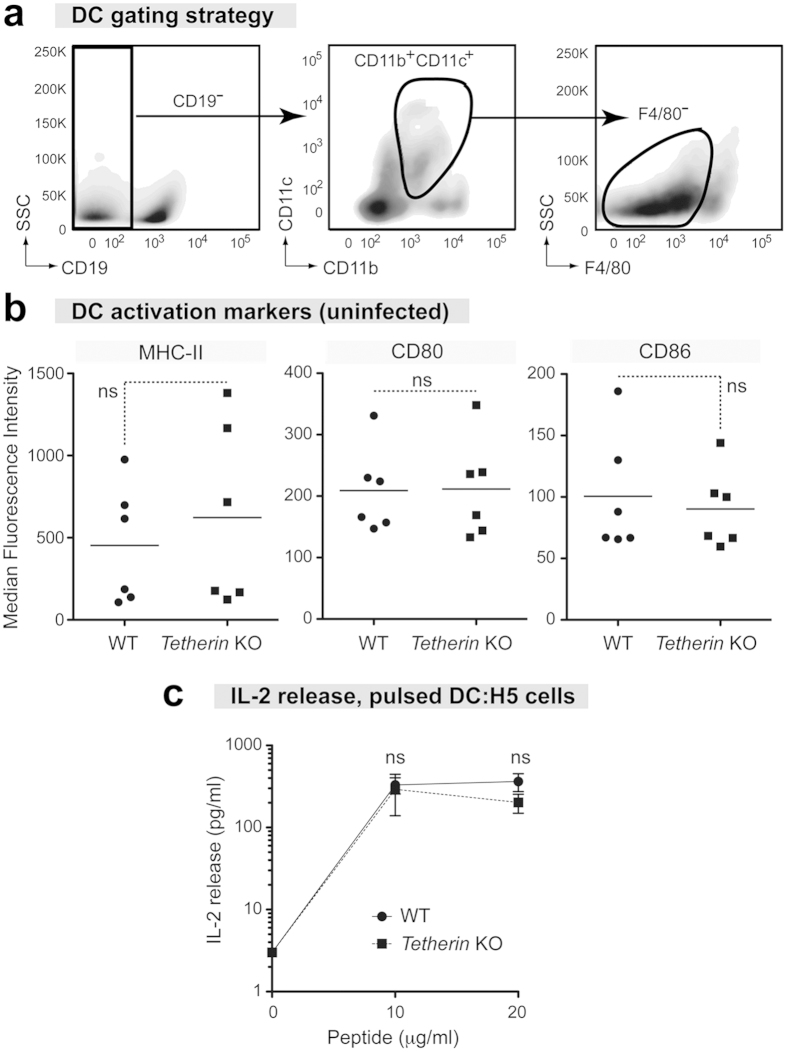
Normal DC phenotypes in Tetherin KO mice. Splenocytes were collected from uninfected WT and Tetherin KO mice and stained for DC activation markers. (**a**) DC gating strategy. CD11c+CD11b+ cells were gated from live CD19- splenocytes and defined as DCs. F4/80 was used to exclude inflammatory monocytes and macrophages. (**b**) Comparison of activation marker expression in DCs from uninfected mice. Median fluorescence intensities of MHC-II, CD80 and CD86 were quantified for WT and Tetherin KO DCs. Lines represent mean values, and each dot corresponds to an individual mouse combined from 2 independent experiments. (**c**) CD4+ T cell stimulation by isolated DCs. Splenocyte DCs enriched by negative magnetic selection were pulsed with 0, 10, 20 or 50 μg/ml of F-MuLV env peptide and cocultured with F-MuLV env-specific CD4+ T cells. IL2 levels in the supernatant were evaluated by ELISA after 2 d. Dots represent mean values and SEM bars from triplicate experiments. Statistical analyses were performed using a 2-tailed Student’s *t* test; ns, not significant at *p* > 0.05.

Conventional myeloid DCs (referred simply as DCs in this study) could modulate both NK and T cell responses^27^. Thus, we next tested whether Tetherin could influence the activity of DCs. We initially determined if Tetherin had an intrinsic effect on DC activation in the absence of infection. Splenocytes from naïve WT and Tetherin KO mice were stained for DC markers and surface proteins indicative of DC maturation. Major histocompatibility complex II (MHC-II) binds antigenic peptide for presentation to CD4+ T cells and its expression on DCs usually increase following infection^35^,^36^. DC activation also results in the induction of costimulatory molecules CD80 and CD86, which work in conjunction with peptide-bound MHC to prime T cell responses^37^. Splenocytes were analyzed by flow cytometry and DCs (CD11c+CD11b+CD19-F4/80-) were evaluated for MHC-II, CD80, and CD86 expression based on median fluorescence intensity (MFI) (Fig. 2a). Expression of MHC-II, CD80, and CD86 were not significantly different between DCs from WT and Tetherin KO mice (Fig. 2b).

Although Tetherin did not affect MHC-II, CD80, and CD86 expression in DCs in uninfected mice, Tetherin may still influence DC function. Thus, splenic DCs from uninfected mice were enriched by negative magnetic selection, pulsed with F-MuLV env_122-141_ peptide and cocultured with H5, an FV env_122−141_-specific CD4+ T cell hybridoma derived from infected B6 mice^38^. IL2 levels in the supernatant after 2 d of DC:H5 co-culture were determined by ELISA. Naïve, peptide-loaded WT and Tetherin KO DCs did not differ in their ability to stimulate IL2 production by the H5 CD4+ T cells (Fig. 2c). Thus, Tetherin did not impact the ability of naïve DCs to stimulate virus-specific CD4+ T cells.

### Tetherin promotes DC activation during acute FV infection

**Figure 3:**
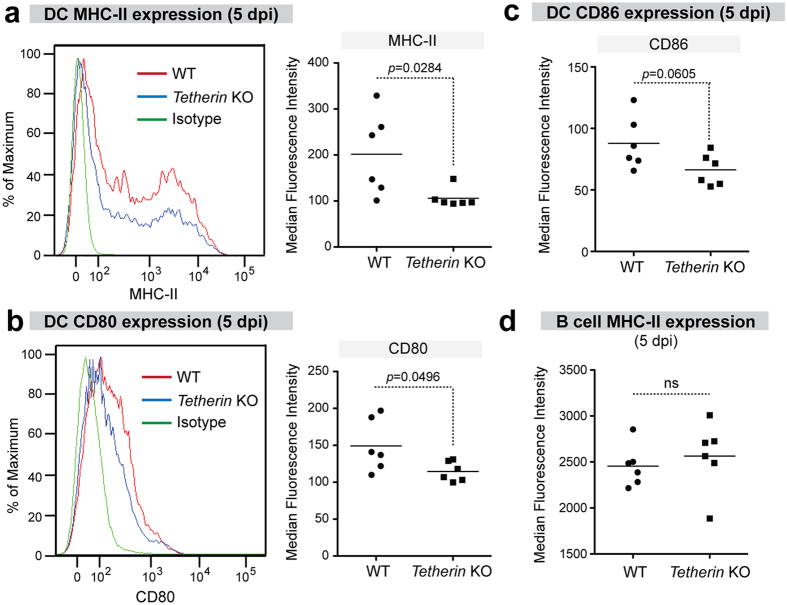
Tetherin promotes DC activation during acute FV infection. WT and Tetherin KO mice were infected with 10^4^ SFFU of FV and at 5 dpi, splenocytes were stained for DC and B cell activation markers. DCs were defined as live CD19-CD11c+CD11b+ cells, B cells as live CD19+ cells. (**a–c**) Splenic DC expression at 5 dpi. Comparison of median fluorescence intensity values for (**a**) MHC-II, (**b**) CD80, and (**c**) CD86 on DCs. Representative histogram plots are shown for MHC-II and CD80 between WT (red) and Tetherin KO (blue) mice. Isotype controls are shown in green. (**d**) MHC-II expression in B cells at 5 dpi. For all panels, lines represent mean values, and each dot corresponds to an individual mouse combined from 2 independent experiments. Differences were evaluated using a 2-tailed unpaired Student’s *t* test; ns, not significant (p > 0.05). Exact *p* values were shown if significant.

Since Tetherin had no effect on DC activation in the absence of infection, we next tested if Tetherin influenced DC activation following FV infection. Splenocytes were collected from FV-infected mice at 5 dpi and gated DCs were analyzed for MHC-II, CD80, and CD86 MFI levels. DCs from infected WT mice showed significantly higher expression of MHC-II and CD80 compared with DCs from infected Tetherin KO mice (Fig. 3a). CD86 expression in DCs was higher in WT versus Tetherin KO mice, but this difference did not quite reach statistical significance (*p* = 0.06). By contrast, MHC-II expression in B cells, which could also serve as antigen presenting cells^39^ were not significantly different between WT and Tetherin KO mice (Fig. 3b). Thus, Tetherin promoted the expression of select proteins involved in antigen presentation in DCs but not in B cells during acute FV infection.

### **Early Tetherin-mediated DC activation correlates with NK cell activity**

**Figure 4:**
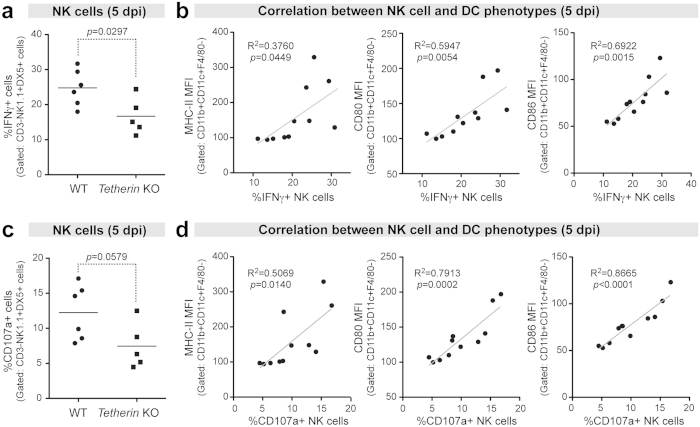
Correlation between Tetherin-dependent DC activation and NK cell activity. Mice were infected with 10^4^ SFFU of FV and at 5 dpi, splenocytes were collected, activated with PMA and ionomycin, then evaluated by flow cytometry. NK cells were gated as CD3-NK1.1+DX5+ cells, and intracellular IFNγ and surface CD107a levels were quantified from these gated NK cells. (**a**) IFNγ+ NK cells in WT versus Tetherin KO spleens. Lines correspond to means and each dot corresponds to individual mice combined from 2 independent experiments. Statistical analyses were performed using a 2-tailed unpaired Student’s t test. Note that one outlier Tetherin KO mouse was excluded due to unusually high levels of NK IFNγ+ cells (51%; 2-sided Dixon Q-test p < 0.05). (**b**) Correlation between NK IFNγ+ cell and DC phenotypes. (*Left*) MHC-II MFI versus IFNγ+ NK cells; (*Middle*) CD80 MFI versus IFNγ+ NK cells. (**c**) CD107a+ NK cells in WT versus Tetherin KO mice were correlated with (**d**) MHC-II and CD80 MFI values. Correlations were evaluated by Pearson r statistics with the best-fit linear regression curves shown in gray lines. Exact *p* values were noted.

We previously showed that Tetherin improved NK cell responses to FV at 14 dpi^16^. However, NK cell responses should already be induced by 1 week post-FV infection^25^. We therefore determined if Tetherin influenced NK cell responses at an earlier time point (5 dpi). Splenocytes from FV-infected mice were stimulated with PMA and ionomycin, stained for NK cell markers (CD3-NK1.1+DX5+), and then analyzed by flow cytometry for expression of IFNγ and CD107a, a marker of NK cell degranulation. A significantly higher percentage of splenic IFNγ+ NK cells were found in WT mice compared to Tetherin KO mice (Fig. 4a). The percentage of IFNγ+ NK cells correlated with DC MHC-II, CD80 and CD86 expression (Fig. 4b). WT mice exhibited higher percentage of CD107a+ cells compared to Tetherin KO mice, but this did not quite reach statistical significance (Fig. 4c; *p* = 0.058). Nevertheless, the percentage of CD107a+ NK cells correlated with DC MHC-II, CD80 and CD86 MFI (Fig. 4d). These data revealed that Tetherin influenced NK cell responses at an early time point following FV infection that correlated with 3 markers of DC activation.

### Tetherin promotes BM IL15 expression

**Figure 5:**
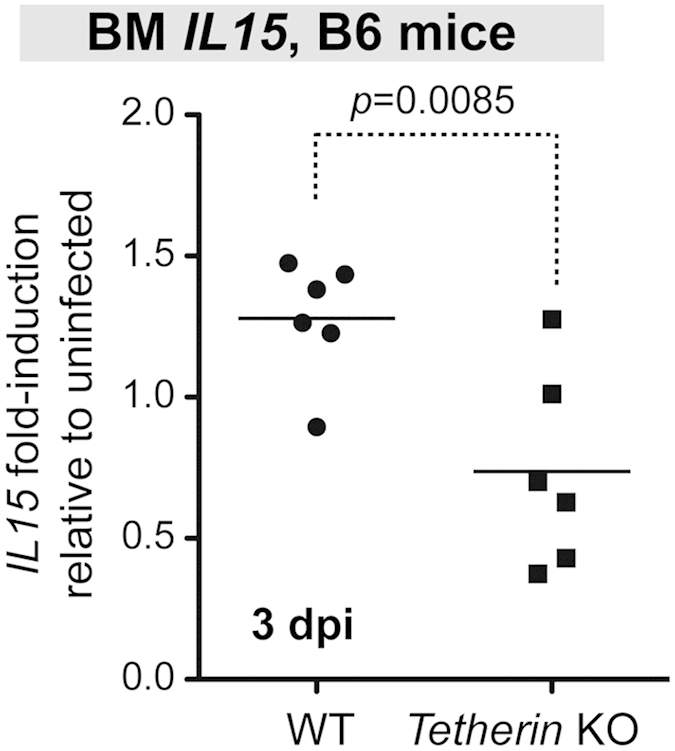
Tetherin promotes BM IL15 expression during acute FV infection. Mice were infected with 10^4^ SFFU of FV and at 3 dpi, RNA from BM cells were extracted for IL15 qPCR. Data were normalized to actin RNA levels, and expressed as fold induction from the BM of an uninfected mouse. Lines correspond to means and each dot corresponds to an individual mouse, and data were combined from 2 independent experiments. Data were analyzed using a 2-tailed unpaired Student’s t-test.

One mechanism by which DCs stimulate NK cells is the production of IL15^40^. IL15 is particularly critical for NK cell development in the BM^41^. We therefore analyzed the levels of IL15 transcripts in BM cells from FV-infected mice by qPCR. IL15 mRNA was expressed at higher levels in the BM of WT mice than in *Tetherin* KO mice at 3 dpi (Fig. 5), but this difference was lost by 5 dpi (*p* > 0.05). Thus, Tetherin transiently enhanced BM IL15 mRNA expression during early retroviral infection.

### Stimulation of FV-specific CD4+ T cells by Tetherin+ versus Tetherin KO DCs

**Figure 6:**
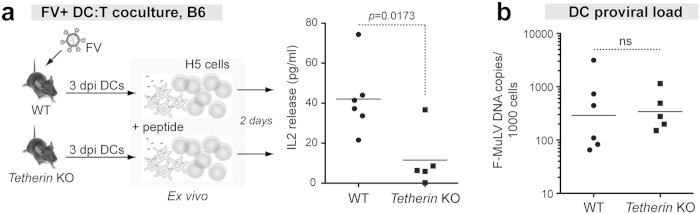
Tetherin promotes DC-mediated antigen presentation to CD4+ T cells. (**a**) DCs were isolated from splenocytes of mice infected with 10^4^ SFFU of FV at 3 dpi by negative magnetic selection. The DCs were co-cultured with an FV-specific CD4+ T cell hybridoma line, H5, and after 2 d, IL2 in the supernatant was quantified by ELISA. DCs from WT and Tetherin KO mice were pulsed with 5 μg/ml F-MuLV env peptide prior to H5 coculture. One Tetherin KO mouse had unusually high splenomegaly (2-sided Grubb’s test, *p *< 0.05) and was excluded. Lines correspond to means, and differences were evaluated using a 2-tailed Mann-Whitney U test. (**b**) Viral infection of DCs. Proviral DNA was quantified from the isolated 3 dpi DCs by qPCR, normalizing to the number of cell equivalents. The lines correspond to geometric means. The dots correspond to individual mice combined from 2 independent experiments. Statistical analyses were performed using a 2-tailed Student’s *t* test; ns, not significant (*p* > 0.05).

Tetherin influenced CD4+ T cell responses at 14 dpi^16^. Our results above demonstrated that Tetherin influenced DC activation and function. Thus, we hypothesized that the impact of Tetherin on CD4+ T cell responses may be preceded by enhanced antigen presentation qualities of Tetherin+ DCs obtained at an earlier time point. WT and Tetherin KO mice were infected with 10^4^ SFFU of FV and at 3 dpi, untouched splenic DCs were isolated by negative selection using magnetic beads. Enriched DCs were cocultured with H5 cells as in Fig. 2d. IL2 release in the DC:H5 cell coculture supernatants were determined by ELISA after 2 days. Isolated DCs from 3 dpi infected WT mice did not stimulate detectable IL2 release from the H5 CD4+ T cells. We therefore pulsed DCs with the F-MuLV env_122-141_ peptide prior to co-culture with H5 cells (Fig. 6a, *left panel*). Notably, WT DCs stimulated IL2 production by H5 cells to a greater degree than Tetherin KO DCs (Fig. 6a, *right panel*). To confirm that the DCs isolated in the mice were exposed to similar amounts of virus, F-MuLV proviral DNA loads in the isolated DCs were quantified. As expected, Tetherin+ and Tetherin KO DCs had similar proviral F-MuLV DNA loads (Fig. 6b). Altogether, the data demonstrate that Tetherin enhanced the antigen presentation capability of DCs to virus-specific CD4+ T cells independent of FV infection levels.

### Tetherin promotes DC activation and function in an Apobec3 KO background

The resistance genes that influence FV infection may exhibit epistasis, such that the presence of one resistance gene may mask the impact of another. In a previous B_1_ test cross of Fv2-susceptible mice, we found that a Tetherin SNP influenced acute FV infection in mice in the mA3/Rfv3 susceptible but not the mA3/Rfv3 resistant background^30^. Thus, Tetherin may have an impact on acute FV replication in an mA3-null background. To test this hypothesis, B6 mA3 KO mice were crossed with Tetherin KO mice to generate mA3/Tetherin double KO (dKO) mice. We then compared FV infection levels, DC activation and antigen presentation capability between B6 mA3 KO (which express Tetherin) and mA3/Tetherin dKO mice.

**Figure 7:**
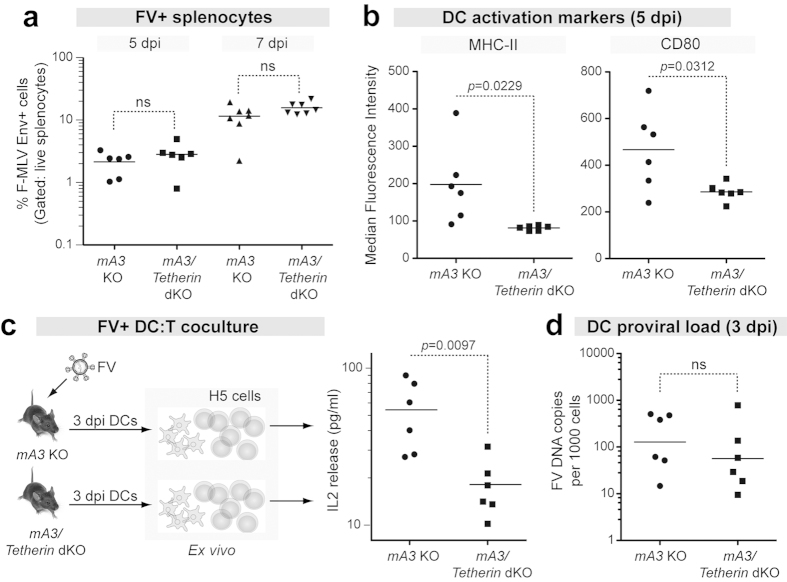
Tetherin promotes DC function in an mA3-null background. mA3 KO (which express Tetherin) and mA3/Tetherin dKO mice were infected with 10^4^ SFFU of FV complex and analyzed at the indicated time points. (**a**) Spleen FV infection levels at 5 and 7 dpi. FV+ cells were determined using a monoclonal antibody against the F-MuLV Env protein. (**b**) DC activation markers. The MHC-II and CD80 MFI values were determined on gated DCs (CD19−CD11b+CD11c+F4/80−) at 5 dpi. (**c**) DC:H5 cocultures. DCs isolated at 3 dpi were coincubated with H5 cells and after 2 days, IL2 release in the supernatant was evaluated by ELISA. (**d**) Proviral DNA load in isolated 3 dpi DCs were determined by qPCR, normalizing to the number of cell equivalents. For all panels, the lines correspond to means and dots correspond to individual mice combined from 2 independent cohorts. The data were analyzed using a 2-tailed unpaired Student’s t test. P values > 0.05 were considered not significant (ns). Exact P values are shown for significant differences.

We first compared FV infection levels between mA3 KO and mA3/Tetherin dKO mice at early time points by flow cytometry. As shown in Fig. 7a, both strains of mice had similar FV infection levels at 5 and 7 dpi. At 5 dpi, DCs of mA3 KO mice expressed higher MHC-II and CD80 MFI levels compared to mA3/Tetherin dKO mice (Fig. 7b). Interestingly, BM cells from mA3 KO mice exhibited higher IL15 expression at 5 dpi compared to mA3/Tetherin dKO mice (Supplementary Fig. 2), in contrast to B6 mice which transiently induced BM IL15 at 3 dpi (Fig. 5). Isolated DCs from 3 dpi mA3 KO mice also more efficiently stimulated IL2 release from virus-specific CD4+ T cells compared to DCs from mA3/Tetherin KO mice (Fig. 7c), despite similar proviral FV DNA loads (Fig. 7d). In contrast to Fig. 6a, a prior peptide pulse was not required to reveal the differences in IL2 release, possibly because of higher FV infection levels in mA3 KO compared to WT mice^42^,^43^. Thus, we confirmed in a second genetic background lacking mA3/Rfv3 that Tetherin significantly promoted DC activation and function during acute retrovirus infection.

## Discussion

Tetherin is a potent retrovirus restriction factor *in vitro* but its impact *in vivo* is still being determined. Using the FV infection model, we previously provided evidence that Tetherin promoted NK cell, CD4+ T cell and CD8+ T cell responses^16^. These stronger cell-mediated immune responses correlated with lower infection levels suggesting that Tetherin-mediated retrovirus control operated by modulating adaptive immunity. Direct inhibition of FV by Tetherin at earlier time points could explain Tetherin’s subsequent immunological effects. Therefore, in the current study, we examined FV infection levels in WT and Tetherin KO mice at earlier acute infection time points. Tetherin had no effect on FV infection levels in the spleen, plasma or isolated DCs from 3 to 7 dpi. Removal of mA3 resulted in increased FV infection, but FV infection levels were still not different between mice with and without Tetherin in this mA3-null background. Our results concur with previous findings using the Mo-MuLV and LP-BM5 infection models that Tetherin did not restrict acute retroviral infection *in vivo*^10^,^11^. Thus, the impact of Tetherin on retrovirus replication was not immediate, as would be expected for an innate restriction factor.

Tetherin’s effects on different arms of cell-mediated immunity, which rely on antigen presenting cells for activation, led us to investigate the possible role of DCs, which serve as critical bridges between innate and adaptive immunity. After confirming that Tetherin did not alter DC phenotypes in uninfected mice, we evaluated DC activation and function during acute FV infection. The antigen presenting function of DCs is dependent on their activation, which is characterized by the upregulation of MHC-II molecules and costimulatory molecules such as CD80 and CD86. Here, we found that DCs from FV+ Tetherin WT mice displayed higher expression of MHC-II and CD80 than DCs from FV+ KO mice. Tetherin+ DCs also more potently stimulated virus-specific CD4+ T cells compared to Tetherin KO DCs *ex vivo* despite similar virus infection levels. The enhanced MHC-II antigen presentation capability of Tetherin+ DCs could explain why WT mice had stronger CD4+ T cell responses compared to Tetherin KO mice^16^. CD4+ T cells play critical roles in the maturation, expansion and function of CD8+ T cells^44^,^45^. Thus, improved MHC-II antigen presentation due to Tetherin activity could indirectly impact CD8+ T cell responses by enhancing CD4+ T cell help. Moreover, NK cells could regulate CD8+ T cell responses against pathogens^46^,^47^. Tetherin influenced NK cell responses as early as 5 dpi, and this phenomenon could also contribute to enhanced CD8+ T cell responses by 14 dpi.

Tetherin-mediated virion aggregation on the cell surface was recently proposed as a mechanism to augment NK cell mediated killing^48^,^49^,^50^. This current model stems from *in vitro* studies showing that cells infected with HIV-1ΔVpu, which could not counteract Tetherin and whose virions aggregate at higher densities on the cell surface, were more susceptible to NK cell mediated antibody-dependent cellular cytotoxicity (ADCC). However, our results from a test cross to evaluate a unique Tetherin single nucleotide polymorphism (SNP) in mice suggest otherwise^16^. Relative to B6 mice, NZW/LacJ (NZW) mice harbor a Tetherin SNP that results in a truncation of the cytoplasmic tail that encodes the endocytosis motif. Endocytosis-defective NZW Tetherin more strongly restricted FV release *in vitro*, consistent with higher retention of virions on the cell surface^30^. Thus, if the virion retention/ADCC model was true, then mice encoding NZW Tetherin should have better NK cell responses than those encoding B6 Tetherin. Surprisingly, we observed the opposite result: mice encoding endocytosis-competent B6 Tetherin demonstrated stronger NK cell-associated immune control of FV *in vivo*^16^. Thus, endocytosis appeared to be critical for Tetherin’s impact on cell-mediated immunity. Interestingly, transfection studies linked Tetherin with HIV-1 Gag accumulation in intracellular compartments^2^,^51^,^52^. Internalization of tethered virions could promote viral sensing by endosomal sensors, resulting in the upregulation of cytokines required for NK cell function such as IL15. Consistent with this theory, we observed higher BM IL15 mRNA levels in WT versus Tetherin KO mice. A recent study found an association between the endosomal sensor TLR3 and cytotoxic NK and CD8+ T cell responses against FV infection^53^. BM-derived DCs from FV+ TLR3 KO mice were less able to stimulate proliferation of CD8+ T cells in culture, whereas splenic DCs from FV+ TLR3 KO mice had lower expression of activation markers. Thus, we hypothesize that Tetherin-mediated virion endocytosis may enhance TLR3 sensing in DCs to promote NK cell responses.

Tetherin-mediated endocytosis of virions could explain the connection between Tetherin and MHC-II antigen presentation found in the current study. First, endosomal recycling of virions could promote sensing to induce MHC-II and co-stimulatory molecule expression. Second, endosomal reuptake may significantly promote the presentation of virion-derived peptides to MHC-II. In contrast to soluble protein antigens that could readily be cleaved by endosomal proteases, virions have macromolecular structures that may render them more protease-resistant^54^. A mechanism that would retain virions in MHC-II antigen-processing compartments, which resemble multivesicular bodies^55^, could therefore increase the amount of viral peptides loaded to MHC-II. Notably, at least 9 H-2^b^-restricted F-MuLV CD4+ T cell epitopes were identified in infected B6 mice^56^. It remains to be determined if this broad epitope diversity could be linked Tetherin. Overall, the results of the current study justify further investigations on Tetherin as a modulator of viral antigen presentation to MHC-II.

Initial models proposed that virus tethering functioned to prevent the release of viruses from infected cells^57^. However, viral tethering may serve a different purpose than just restricting virus release. Tetherin induced NF-κB when tethering HIV-1^31^,^32^,^33^,^34^, suggesting that Tetherin could act as a viral sensor. As mouse Tetherin could not activate NF-κB, additional immunological phenotypes may be altered by human Tetherin *in vivo*. In this study, we found that human Tetherin activated NF-κB in mouse cells, suggesting that transgenic mice encoding human Tetherin may be useful for probing these additional immunological effects *in vivo*. Interestingly, mouse Tetherin enhanced type I IFN production by a specialized subset of cells known as plasmacytoid dendritic cells (pDC), when stimulated with virus *in vitro*^11^. This appears to contradict data revealing that the interaction between human Tetherin and ILT7 lowers pDC activation^58^. However, a recent report revealed that HIV-1 Vpu may facilitate Tetherin-mediated suppression of pDC activation by targeting Tetherin outside viral assembly sites to interact with ILT7^59^. Studies are now underway to determine if Tetherin influences pDC function and type I IFN production during retrovirus infection *in vivo*.

In conclusion, we provide evidence that enhanced myeloid DC activation and function underlie the observed connection between Tetherin and retrovirus cell-mediated immune responses. Furthermore, the data raise the possibility that Tetherin’s primary role during retrovirus infection may be immunological, as Tetherin-mediated inhibition of acute FV infection was not observed. Data from the current study and previous work on an endocytosis-defective Tetherin allele^16^,^30^ support a model whereby viral tethering triggers the endocytic recycling of tethered virus for enhanced TLR sensing and antigen presentation. Specific molecular details of this proposed pathway remain to be unraveled. Answering these emerging mechanistic questions on Tetherin immunobiology could have important implications in boosting cell-mediated immune responses against pathogenic retrovirus infections including HIV-1.

## Methods

### Mice

B6 mice (*Fv1*^b/b^ *Fv2*^r/r^ *H-2*^b/b^ mA3/*Rfv3*^r/r^) were purchased from The Jackson Laboratory. mA3 KO mice were generated from the XN450 gene-trap embryonic stem cell line (BayGenomics) and backcrossed for nine generations into B6^60^. Tetherin KO mice were directly generated in the B6 genetic background^10^. mA3 KO mice were crossed with Tetherin KO mice and resulting F_1_ progeny were crossed to each other to generate mA3/Tetherin double KO (dKO) mice. Mice used in this study ranged from 8 to 12 weeks of age. Mice were handled in accordance with the regulations of the National Institutes of Health Guide for the Care and Use of Laboratory Animals. The procedures include tail clipping at <3 weeks of age for genotyping, breeding, infections with FV via the intravenous route and terminal euthanasia involving carbon dioxide inhalation followed by cervical dislocation. Infections were performed under isoflurane anesthesia with regular monitoring and all efforts were made to minimize suffering of the animals. This study was approved by the University of Colorado Institutional Animal Care and Use Committee Permit Number B-89712(08)1E.

### Cell culture

H5, a CD4+ T cell hybridoma line specific to the F-MuLV env_122−141_ peptide^38^, was cultured in Iscove’s Modified Dulbecco’s Medium (IMDM) (Life Technologies) containing 5% Fetal Bovine Serum (Gemini) and penicillin/streptomycin/glutamine (Mediatech).

### **NF-**κ**B activation assay**

Mouse NIH3T3 cells were transfected with 500 ng of an NF-kB firefly luciferase reporter plasmid^31^, 200 ng of a Renilla luciferase expression plasmid, and 500 ng of either vector control (p3xFLAG; Sigma), mouse Tetherin^30^ or human Tetherin^31^ expression plasmid (generously provided by Drs. Rui Galao and Stuart Neil), using the TransIT-LT1 transfection reagent (Mirus Bio). After 48 h, cells were lysed, and lysates were treated with luciferase substrate reagents from a Dual-Luciferase Reporter Assay Kit (Promega). Bioluminescence from firefly and Renilla luciferase activity were detected sequentially using a VictorX5 plate reader (Perkin Elmer). The firefly luciferase signal was normalized to the Renilla luciferase signal to control for transfection efficiency.

### FV infection

Mice were infected intravenously with 10,000 spleen focus-forming units (SFFU) of FV, a complex composed of B-tropic F-MuLV and spleen focus-forming virus, SFFV. The virus stock lacks lactate-dehydrogenase elevating virus^61^,^62^. All virological assays used in this study were specific for the F-MuLV helper virus, which is critical for FV replication. The virus stock was prepared and titered in BALB/c mice^63^.

### Plasma Viral Load analysis

Plasma viral load was measured by quantitative real-time PCR (qPCR)^64^. RNA was extracted from 50 μl plasma using the Qiagen RNAeasy kit and eluted in 100 μl water. RNA (10 μl) was added to 1 × 1-step TaqMan Reverse Transcriptase PCR reaction mix (Applied Biosystems) along with 10 pmol of the following primers and probe: F-MuLV sense, 5′-GGACAGAAACTACCGCCCTG-3′; F-MuLV antisense, 5′-ACAA- CCTCAGACAACGAAGTAAGA-3′; and F-MuLV probe, FAM-TCGCCACCCAGCAGTTTCAGCAGC-TAMRA. Real-time PCR was performed in a Bio-Rad CFX96 cycler using these thermocycling conditions: 48 °C for 15 min, 95 °C for 10 min, 40 cycles of 95 °C for 15 s, 60 °C for 1 min. T7-transcribed F-MuLV RNA standards were used to interpolate absolute F-MuLV RNA copy numbers in the plasma.

### Immunophenotyping

Splenocytes were disaggregated through a 100 μm nylon filter to generate a single cell suspension. Bone marrow cells were collected from at least 2 femurs. Splenocytes and BM cells from naïve and infected mice were stained with the F-MuLV Env gp70-specific mAb 720 for 1 h, then co-stained with: CD11b-V500 (M1/70) (BD Biosciences), CD11c-PE-Cy7 (N418) (eBioscience); CD19-allophycocyanin-H7 (6D5) (Biolegend); F4/80-PerCP-Cy5.5 (eBioscience); MHC class II (I-A/I-E)-Alexa Fluor 700 (M5/114.15.2) (eBioscience); CD80-FITC (16-10A1) (eBioscience); CD86-Brilliant Violet 421 (GL-1) (Biolegend); and anti-mouse IgG-allophycocyanin (Columbia Biosciences) for 30 min. Conventional dendritic cells (DCs) were classified as CD19-CD11b+CD11c+F4/80-. MHC class II (MHC-II) was not used to gate DCs as MHC-II is downregulated during FV infection^29^ and some mouse splenic DC subsets do not express MHC-II^65^. Since CD11c can be expressed by B cells^66^ and inflammatory monocytes^67^, the CD19 and F4/80 markers were used to exclude these cell subpopulations, respectively.

A separate aliquot of splenocytes (4 × 10^6^) were treated with RBC lysis buffer (eBioscience), stimulated with PMA (25 ng/ml) and Ionomycin (0.7 ug/ml) (Sigma-Aldrich), and stained with CD107a-PE-Cy7 (1D4B) (BD Biosciences) for 5 h at 37 °C and 5% CO_2_. The cells were treated with Golgi Plug (BD Biosciences) for the final 4 h of incubation. The cells were then co-stained with either NK-1.1-allophycocyanin (PK136), CD49b-FITC (DX5), and CD3-AlexaFluor700 (17A2) (BD Bioscience), or with CD8α-FITC (53–6.7) and CD4-PE-CF594 (RM4-5) (BD Biosciences) for 30 min. The cells were permeabilized in Perm/Fix buffer (BD Biosciences), and stained with IFNγ-PE (XMG1.2) (BD Biosciences). All cells were fixed in 1% paraformaldehyde before analysis with an LSR-II flow cytometer (BD Biosciences). Up to 500,000 events per sample was captured for DC analysis and up to 250,000 events per sample was captured for NK cell and T cell anlaysis. FlowJo software (Treestar) was used for data analysis. Isotype controls were used for gating. Spleen and bone marrow cells from uninfected mice stained with mAb720 were used to establish gates for FV-infected cells.

### DC and CD4+ T cell coculture

Splenocytes were collected from uninfected or FV-infected mice at 3 dpi and passed through a 100 μm nylon filter. Cells were then treated with RBC lysis buffer (eBioscience). A pan-dendritic cell negative selection kit (Miltenyi 130-100-875) was used for Magnetic-Activated Cell Sorting (MACS) of DCs from the splenocytes. MACS separation was performed according to the manufacturer’s instructions. 10^5^ DCs from each mouse were aliquoted into wells of a 96-well tissue culture plate. DCs were pulsed with 0, 5, 10, 20, or 50 μg/ml of F-MuLV env_122−141_ peptide (DEPLTSLTPRCNTAWNRLKL) (Genscript) diluted in water for 1 h. Excess peptide was washed from the cells. 4 × 10^5 ^H5 cells^38^ were added to each DC culture for a final volume of 200 μl. After 2 d of coculture at 37 °C and 5% CO_2_, supernatants were collected and used for IL2 ELISA.

### IL2 ELISA

IL2 in supernatants collected from coculture experiments were quantified using an IL2 ELISA kit (eBioscience 88-7024-22). Experiments were performed according to the manufacturers’ instructions.

### IL15 expression

RNA was isolated from BM using the Qiagen RNAeasy kit. cDNA synthesis was performed using the QuantiTect Reverse Transcription Kit (Qiagen) and the product was diluted 1:10 in water for a final volume of 200 μl. Diluted cDNA (10 μl) was added to 1× QuantiTect SYBR Green PCR master mix (Qiagen) and 5 pmol of the following primers: IL15.for, CATTTTGGGCTGTGTCAGTG; IL15.rev, CATTTTGGGCTGTGTCAGTG. The following thermocycling conditions were used: 95 °C for 10 min, 40 cycles of 95 °C for 15 s, 60 °C for 1 min. Melt curve analysis and gel electrophoresis were used to verify the purity of the amplicon. Fold inductions were normalized to β-actin and calculated using the formula: Fold induction = 2^(−ΔΔCt), where ΔΔCt = [Ct_IL15_(Infected) − Ct_actin_(Infected)] − [Ct_IL15_(Naïve) − Ct_actin_(Naïve)].

### FV proviral load

FV proviral loads in enriched DCs were determined by qPCR^68^. DNA was extracted from enriched DCs from the spleen using the Qiagen DNAeasy Blood and Tissue kit, and 100 ng DNA was added to 1× Taqman Gene Expression Master Mix (Qiagen) along with 10 pmol F-MuLV-specific primers and probe described above. Absolute F-MuLV DNA copy numbers were interpolated using F-MuLV DNA standards. FV DNA copies per cell was determined by also measuring mA3 DNA copies, with 2 *mA3* copies = 1 cell. 10 pmol of the following primers and probe were added to the same master mix for duplex PCR: mA3.sense, 5′-ATCCTCTTCCTTGATAAGATTCGGTCCATG-3′; mA3.antisense, 5′-GATCCCTGATTGCCACAGAGAACAC-3′; mA3.probe, HEX-ATC TACACCTCCCGCCTGTATTTCCACT-TAMRA. mA3 DNA standards were used to interpolate absolute mA3 DNA copy numbers. The reaction was performed under these thermocycling conditions: 95 °C for 10 min, 40 cycles of 95 °C for 15 s, 60 °C for 1 min.

### Statistical analysis

Statistical analyses were performed using Prism 5.0 (Graphpad). Two-tailed Student’s *t* test was used for 2-group comparisons. For datasets with skewed distribution based on the Kolmogorov-Smirnov normality test (p < 0.05), a nonparametric 2-tailed Mann-Whitney *U* test was performed. Correlations were computed using Pearson’s r. *P* values <0.05 were considered statistically significant.

## Additional Information

**How to cite this article**: Li, S. X. *et al.* Tetherin/BST-2 promotes dendritic cell activation and function during acute retrovirus infection. *Sci. Rep.* **6**, 20425; doi: 10.1038/srep20425 (2016).

## Supplementary Material

Supplementary Information

## References

[b1] Blanco-Melo D., Venkatesh S. & Bieniasz P. D. Intrinsic cellular defenses against human immunodeficiency viruses. Immunity 37, 399–411 (2012). 22999946 10.1016/j.immuni.2012.08.013 PMC3912573

[b2] Neil S. J., Zang T. & Bieniasz P. D. Tetherin inhibits retrovirus release and is antagonized by HIV-1 Vpu. Nature 451, 425–430 (2008). 18200009 10.1038/nature06553

[b3] Van Damme N. *et al.* The interferon-induced protein BST-2 restricts HIV-1 release and is downregulated from the cell surface by the viral Vpu protein. Cell Host Microbe 3, 245–252 (2008). 18342597 10.1016/j.chom.2008.03.001 PMC2474773

[b4] Perez-Caballero D. *et al.* Tetherin inhibits HIV-1 release by directly tethering virions to cells. Cell 139, 499–511 (2009). 19879838 10.1016/j.cell.2009.08.039 PMC2844890

[b5] Casartelli N. *et al.* Tetherin restricts productive HIV-1 cell-to-cell transmission. PLoS Pathog 6, e1000955 (2010). 20585562 10.1371/journal.ppat.1000955 PMC2887479

[b6] Pais-Correia A. M. *et al.* Biofilm-like extracellular viral assemblies mediate HTLV-1 cell-to-cell transmission at virological synapses. Nat Med 16, 83–89 (2010). 20023636 10.1038/nm.2065

[b7] Dietrich I. *et al.* Feline tetherin efficiently restricts release of feline immunodeficiency virus but not spreading of infection. J Virol 85, 5840–5852 (2011). 21490095 10.1128/JVI.00071-11 PMC3126296

[b8] Andrew A. & Strebel K. The interferon-inducible host factor bone marrow stromal antigen 2/tetherin restricts virion release, but is it actually a viral restriction factor? J Interferon Cytokine Res 31, 137–144 (2011). 21166593 10.1089/jir.2010.0108 PMC3021358

[b9] Jolly C., Booth N. J. & Neil S. J. Cell-cell spread of human immunodeficiency virus type 1 overcomes tetherin/BST-2-mediated restriction in T cells. J Virol 84, 12185–12199 (2010). 20861257 10.1128/JVI.01447-10 PMC2976402

[b10] Liberatore R. A. & Bieniasz P. D. Tetherin is a key effector of the antiretroviral activity of type I interferon *in vitro* and *in vivo*. Proc Natl Acad Sci USA 108, 18097–18101 (2011). 22025715 10.1073/pnas.1113694108 PMC3207693

[b11] Swiecki M., Wang Y., Gilfillan S., Lenschow D. J. & Colonna M. Cutting edge: paradoxical roles of BST2/tetherin in promoting type I IFN response and viral infection. J Immunol 188, 2488–2492 (2012). 22327075 10.4049/jimmunol.1103145 PMC3522186

[b12] Jones P. H., Mahauad-Fernandez W. D., Madison M. N. & Okeoma C. M. BST-2/tetherin is overexpressed in mammary gland and tumor tissues in MMTV-induced mammary cancer. Virology 444, 124–139 (2013). 23806386 10.1016/j.virol.2013.05.042 PMC4026021

[b13] Ho O. & Green W. R. Cytolytic CD8+ T cells directed against a cryptic epitope derived from a retroviral alternative reading frame confer disease protection. J Immunol 176, 2470–2475 (2006). 16456007 10.4049/jimmunol.176.4.2470

[b14] Halemano K., Barrett B. S., Heilman K. J., Morrison T. E. & Santiago M. L. Requirement for Fc effector mechanisms in the APOBEC3/Rfv3-dependent neutralizing antibody response. J Virol 89, 4011–4014 (2015). 25589647 10.1128/JVI.03399-14 PMC4403405

[b15] Halemano K. *et al.* Immunoglobulin somatic hypermutation by APOBEC3/Rfv3 during retroviral infection. Proc Natl Acad Sci USA 111, 7759–7764 (2014). 24821801 10.1073/pnas.1403361111 PMC4040588

[b16] Li S. X. *et al.* Tetherin Promotes the Innate and Adaptive Cell-Mediated Immune Response against Retrovirus Infection *In Vivo*. J Immunol 193, 306–316 (2014). 24872193 10.4049/jimmunol.1400490 PMC4163935

[b17] Halemano K. *et al.* Humoral immunity in the Friend retrovirus infection model. Immunol Res 55, 249–260 (2013). 22961660 10.1007/s12026-012-8370-y PMC4003891

[b18] Halemano K. *et al.* Fv1 restriction and retrovirus vaccine immunity in Apobec3-deficient 129P2 mice. PloS ONE 8, e60500 (2013). 23533681 10.1371/journal.pone.0060500 PMC3606284

[b19] Van der Gaag H. C. & Axelrad A. A. Friend virus replication in normal and immunosuppressed C57BL/6 mice. Virology 177, 837–839 (1990). 1973551 10.1016/0042-6822(90)90561-5

[b20] Hasenkrug K. J. Lymphocyte deficiencies increase susceptibility to friend virus-induced erythroleukemia in Fv-2 genetically resistant mice. J Virol 73, 6468–6473 (1999). 10400741 10.1128/jvi.73.8.6468-6473.1999 PMC112728

[b21] Joedicke J. J., Zelinskyy G., Dittmer U. & Hasenkrug K. J. CD8+ T cells are essential for controlling acute friend retrovirus infection in C57BL/6 mice. J Virol 88, 5200–5201 (2014). 24707025 10.1128/JVI.00312-14 PMC3993795

[b22] Zelinskyy G. *et al.* Virus-specific CD8+ T cells upregulate programmed death-1 expression during acute friend retrovirus infection but are highly cytotoxic and control virus replication. J Immunol 187, 3730–3737 (2011). 21873525 10.4049/jimmunol.1101612 PMC3402334

[b23] Hasenkrug K. J., Brooks D. M. & Dittmer U. Critical role for CD4(+) T cells in controlling retrovirus replication and spread in persistently infected mice. J Virol 72, 6559–6564 (1998). 9658100 10.1128/jvi.72.8.6559-6564.1998 PMC109830

[b24] Pike R. *et al.* Race between retroviral spread and CD4+ T-cell response determines the outcome of acute Friend virus infection. J Virol 83, 11211–11222 (2009). 19692462 10.1128/JVI.01225-09 PMC2772778

[b25] Littwitz E., Francois S., Dittmer U. & Gibbert K. Distinct roles of NK cells in viral immunity during different phases of acute Friend retrovirus infection. Retrovirology 10, 127 (2013). 24182203 10.1186/1742-4690-10-127 PMC3826539

[b26] Zelinskyy G., Balkow S., Schimmer S., Werner T., Simon M. M. & Dittmer U. The level of friend retrovirus replication determines the cytolytic pathway of CD8+ T-cell-mediated pathogen control. J Virol 81, 11881–11890 (2007). 17728236 10.1128/JVI.01554-07 PMC2168789

[b27] Fernandez N. C. *et al.* Dendritic cells directly trigger NK cell functions: cross-talk relevant in innate anti-tumor immune responses *in vivo*. Nat Med 5, 405–411 (1999). 10202929 10.1038/7403

[b28] Walzer T., Dalod M., Robbins S. H., Zitvogel L. & Vivier E. Natural-killer cells and dendritic cells: “l’union fait la force”. Blood 106, 2252–2258 (2005). 15933055 10.1182/blood-2005-03-1154

[b29] Balkow S., Krux F., Loser K., Becker J. U., Grabbe S. & Dittmer U. Friend retrovirus infection of myeloid dendritic cells impairs maturation, prolongs contact to naive T cells, and favors expansion of regulatory T cells. Blood 110, 3949–3958 (2007). 17699743 10.1182/blood-2007-05-092189

[b30] Barrett B. S., Smith D. S., Li S. X., Guo K., Hasenkrug K. J. & Santiago M. L. A single nucleotide polymorphism in tetherin promotes retrovirus restriction *in vivo*. PLoS Pathog 8, e1002596 (2012). 22457621 10.1371/journal.ppat.1002596 PMC3310811

[b31] Galao R. P., Le Tortorec A., Pickering S., Kueck T. & Neil S. J. Innate sensing of HIV-1 assembly by Tetherin induces NFkappaB-dependent proinflammatory responses. Cell Host Microbe 12, 633–644 (2012). 23159053 10.1016/j.chom.2012.10.007 PMC3556742

[b32] Tokarev A., Suarez M., Kwan W., Fitzpatrick K., Singh R. & Guatelli J. Stimulation of NF-kappaB activity by the HIV restriction factor BST2. J Virol 87, 2046–2057 (2013). 23221546 10.1128/JVI.02272-12 PMC3571454

[b33] Cocka L. J. & Bates P. Identification of alternatively translated Tetherin isoforms with differing antiviral and signaling activities. PLoS Pathog 8, e1002931 (2012). 23028328 10.1371/journal.ppat.1002931 PMC3460627

[b34] Billcliff P. G., Rollason R., Prior I., Owen D. M., Gaus K. & Banting G. CD317/tetherin is an organiser of membrane microdomains. J Cell Sci 126, 1553–1564 (2013). 23378022 10.1242/jcs.112953 PMC3647434

[b35] Cella M., Engering A., Pinet V., Pieters J. & Lanzavecchia A. Inflammatory stimuli induce accumulation of MHC class II complexes on dendritic cells. Nature 388, 782–787 (1997). 9285591 10.1038/42030

[b36] Pierre P. *et al.* Developmental regulation of MHC class II transport in mouse dendritic cells. Nature 388, 787–792 (1997). 9285592 10.1038/42039

[b37] Mellman I. & Steinman R. M. Dendritic cells: specialized and regulated antigen processing machines. Cell 106, 255–258 (2001). 11509172 10.1016/s0092-8674(01)00449-4

[b38] Young G. R., Ploquin M. J., Eksmond U., Wadwa M., Stoye J. P. & Kassiotis G. Negative selection by an endogenous retrovirus promotes a higher-avidity CD4+ T cell response to retroviral infection. PLoS Pathog 8, e1002709 (2012). 22589728 10.1371/journal.ppat.1002709 PMC3349761

[b39] Schultz K. R., Klarnet J. P., Gieni R. S., HayGlass K. T. & Greenberg P. D. The role of B cells for *in vivo* T cell responses to a Friend virus-induced leukemia. Science 249, 921–923 (1990). 2118273 10.1126/science.2118273

[b40] Lucas M., Schachterle W., Oberle K., Aichele P. & Diefenbach A. Dendritic cells prime natural killer cells by trans-presenting interleukin 15. Immunity 26, 503–517 (2007). 17398124 10.1016/j.immuni.2007.03.006 PMC2084390

[b41] Puzanov I. J., Bennett M. & Kumar V. IL-15 can substitute for the marrow microenvironment in the differentiation of natural killer cells. J Immunol 157, 4282–4285 (1996). 8906800

[b42] Smith D. S. *et al.* Noninfectious retrovirus particles drive the APOBEC3/Rfv3 dependent neutralizing antibody response. PLoS Pathog 7, e1002284 (2011). 21998583 10.1371/journal.ppat.1002284 PMC3188525

[b43] Santiago M. L., Benitez R. L., Montano M., Hasenkrug K. J. & Greene W. C. Innate retroviral restriction by Apobec3 promotes antibody affinity maturation *in vivo*. J Immunol 185, 1114–1123 (2010). 20566830 10.4049/jimmunol.1001143 PMC3024598

[b44] Khanolkar A., Fuller M. J. & Zajac A. J. CD4 T cell-dependent CD8 T cell maturation. J Immunol 172, 2834–2844 (2004). 14978084 10.4049/jimmunol.172.5.2834

[b45] Janssen E. M., Lemmens E. E., Wolfe T., Christen U., von Herrath M. G. & Schoenberger S. P. CD4+ T cells are required for secondary expansion and memory in CD8+ T lymphocytes. Nature 421, 852–856 (2003). 12594515 10.1038/nature01441

[b46] Vankayalapati R. *et al.* NK cells regulate CD8+ T cell effector function in response to an intracellular pathogen. J Immunol 172, 130–137 (2004). 14688318 10.4049/jimmunol.172.1.130

[b47] Waggoner S. N., Cornberg M., Selin L. K. & Welsh R. M. Natural killer cells act as rheostats modulating antiviral T cells. Nature 481, 394–398 (2012). 22101430 10.1038/nature10624 PMC3539796

[b48] Arias J. F. *et al.* Tetherin antagonism by Vpu protects HIV-infected cells from antibody-dependent cell-mediated cytotoxicity. Proc Natl Acad Sci USA 111, 6425–3430 (2014). 24733916 10.1073/pnas.1321507111 PMC4035966

[b49] Alvarez R. A. *et al.* HIV-1 Vpu antagonism of tetherin inhibits antibody-dependent cellular cytotoxic responses by natural killer cells. J Virol 88, 6031–6046 (2014). 24623433 10.1128/JVI.00449-14 PMC4093850

[b50] Veillette M. *et al.* The HIV-1 gp120 CD4-bound conformation is preferentially targeted by antibody-dependent cellular cytotoxicity-mediating antibodies in sera from HIV-1-infected individuals. J Virol 89, 545–551 (2015). 25339767 10.1128/JVI.02868-14 PMC4301108

[b51] Neil S. J., Eastman S. W., Jouvenet N. & Bieniasz P. D. HIV-1 Vpu promotes release and prevents endocytosis of nascent retrovirus particles from the plasma membrane. PLoS Pathog 2, e39 (2006). 16699598 10.1371/journal.ppat.0020039 PMC1458960

[b52] Miyakawa K. *et al.* BCA2/Rabring7 promotes tetherin-dependent HIV-1 restriction. PLoS Pathog 5, e1000700 (2009). 20019814 10.1371/journal.ppat.1000700 PMC2788703

[b53] Gibbert K. *et al.* Friend retrovirus drives cytotoxic effectors through Toll-like receptor 3. Retrovirology 11, 1 (2014). 25539593 10.1186/s12977-014-0126-4 PMC4299798

[b54] Eisenlohr L. C. Alternative generation of MHC class II-restricted epitopes: not so exceptional? Mol Immunol 55, 169–171 (2013). 23200635 10.1016/j.molimm.2012.10.020 PMC3610803

[b55] Chu H. *et al.* Tetherin/BST-2 is essential for the formation of the intracellular virus-containing compartment in HIV-infected macrophages. Cell Host Microbe 12, 360–372 (2012). 22980332 10.1016/j.chom.2012.07.011 PMC3444820

[b56] Messer R. J., Lavender K. J. & Hasenkrug K. J. Mice of the resistant H-2(b) haplotype mount broad CD4(+) T cell responses against 9 distinct Friend virus epitopes. Virology 456–457, 139–144 (2014). 10.1016/j.virol.2014.03.012 PMC4044627 24889233

[b57] Gottlinger H. G. HIV/AIDS: virus kept on a leash. Nature 451, 406–408 (2008). 18200012 10.1038/nature06364

[b58] Cao W. *et al.* Regulation of TLR7/9 responses in plasmacytoid dendritic cells by BST2 and ILT7 receptor interaction. J Exp Med 206, 1603–1614 (2009). 19564354 10.1084/jem.20090547 PMC2715090

[b59] Bego M. G., Cote E., Aschman N., Mercier J., Weissenhorn W. & Cohen E. A. Vpu Exploits the cross-talk between BST2 and the ILT7 receptor to suppress anti-HIV-1 responses by plasmacytoid dendritic cells. PLoS Pathog 11, e1005024 (2015). 26172439 10.1371/journal.ppat.1005024 PMC4501562

[b60] Barrett B. S. *et al.* Reassessment of murine APOBEC1 as a retrovirus restriction factor *in vivo*. Virology 468–470C, 601–608 (2014). 10.1016/j.virol.2014.09.006 PMC4430097 25303118

[b61] Robertson S. J. *et al.* Suppression of acute anti-friend virus CD8+ T-cell responses by coinfection with lactate dehydrogenase-elevating virus. J Virol 82, 408–418 (2008). 17959678 10.1128/JVI.01413-07 PMC2224392

[b62] Marques R., Antunes I., Eksmond U., Stoye J., Hasenkrug K. & Kassiotis G. B lymphocyte activation by coinfection prevents immune control of friend virus infection. J Immunol 181, 3432–3440 (2008). 18714015 10.4049/jimmunol.181.5.3432 PMC2655735

[b63] Santiago M. L. *et al.* Apobec3 encodes Rfv3, a gene influencing neutralizing antibody control of retrovirus infection. Science 321, 1343–1346 (2008). 18772436 10.1126/science.1161121 PMC2701658

[b64] Harper M. S. *et al.* IFN-alpha treatment inhibits acute Friend retrovirus replication primarily through the antiviral effector molecule Apobec3. J Immunol 190, 1583–1590 (2013). 23315078 10.4049/jimmunol.1202920 PMC3654153

[b65] Tan J. K., Quah B. J., Griffiths K. L., Periasamy P., Hey Y. Y. & O’Neill H. C. Identification of a novel antigen cross-presenting cell type in spleen. J Cell Mol Med 15, 1189–1199 (2011). 20477902 10.1111/j.1582-4934.2010.01089.x PMC3822631

[b66] Rubtsova K., Rubtsov A. V., van Dyk L. F., Kappler J. W. & Marrack P. T-box transcription factor T-bet, a key player in a unique type of B-cell activation essential for effective viral clearance. Proc Natl Acad Sci USA 110, E3216–3224 (2013). 23922396 10.1073/pnas.1312348110 PMC3752276

[b67] Drutman S. B., Kendall J. C. & Trombetta E. S. Inflammatory spleen monocytes can upregulate CD11c expression without converting into dendritic cells. J Immunol 188, 3603–3610 (2012). 22442444 10.4049/jimmunol.1102741 PMC4594880

[b68] Li S. X. *et al.* Ribonuclease L is not critical for innate restriction and adaptive immunity against Friend retrovirus infection. Virology 443, 134–142 (2013). 23725696 10.1016/j.virol.2013.05.009 PMC3866974

