# Supplementary material for: Tetherin/BST-2 promotes dendritic cell activation and function during acute retrovirus infection
Source: Sci Rep. 2016 Feb 5;6:20425. doi: 10.1038/srep20425 (PMC4742778; doi:10.1038/srep20425)
Supplement: Supplementary Information [file srep20425-s1.pdf]

Supplementary Information

Tetherin/BST-2 promotes dendritic cell activation and function during acute retrovirus infection

Sam X. Li*[,†], Bradley S. Barrett*, Kejun Guo*, George Kassiotis[‡], Kim J. Hasenkrug[§], Ulf Dittmer[¶], Kathrin Gibbert[¶], and Mario L. Santiago*[,†]

*Department of Medicine, Aurora, University of Colorado Denver, CO 80045, USA
[†]Department of Immunology and Microbiology, University of Colorado Denver, Aurora, CO 80045, USA
[‡]The Francis Crick Institute, Mill Hill Laboratory, London, UK and Faculty of Medicine, Department of Medicine, Imperial College London, UK
[§]Rocky Mountain Laboratories, National Institutes of Allergy and Infectious Diseases, National Institutes of Health, Hamilton, MT 59840, USA
[¶]Institute for Virology, University Hospital in Essen, University of Duisburg-Essen, Essen, Germany
*Correspondence should be addressed to M.L.S. (mario.santiago@ucdenver.edu)

Supplementary Figure 1

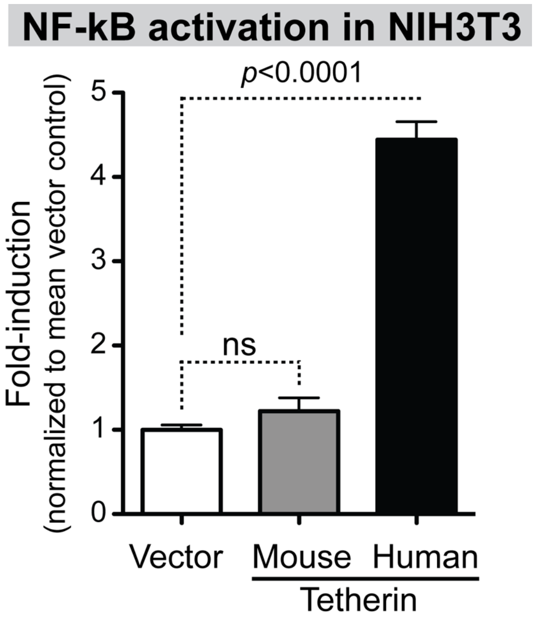

Supplementary Figure 1 | NF-κB activation by human versus mouse Tetherin in mouse NIH3T3 cells. NIH3T3 cells were transfected with an NF-κB luciferase reporter plasmid, along with a mouse or human Tetherin expression plasmid or vector control. After 2 days, cells were lysed and analyzed for luciferase activity. NF-κB induction is expressed as fold change from the mean values using vector control. Bars correspond to mean and error bars correspond to standard deviations from triplicate transfections. Statistics were done using a one-way Analysis of Variance followed by a Dunnett's multiple comparison. ns, not significant.

Supplementary Figure 2

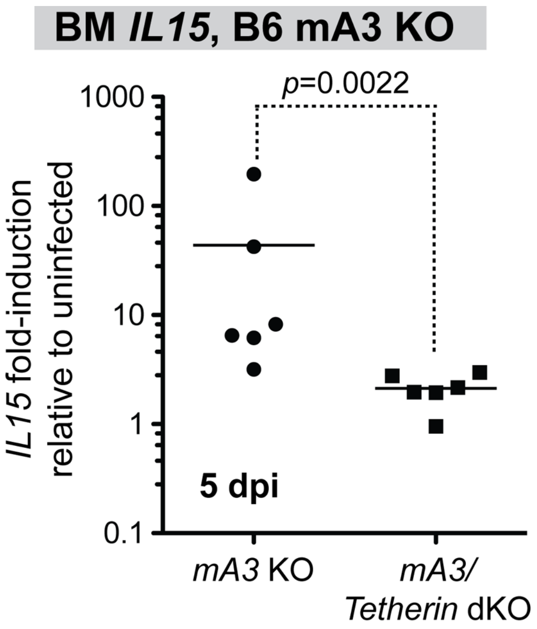

Supplementary Figure 2 | Tetherin promotes BM IL15 expression during acute FV infection of mA3/Rfv3-null mice. Mice were infected with 10^4 SFFU of FV and at 5 dpi, RNA from BM cells were extracted for IL15 qPCR. Data were normalized to actin RNA levels, and expressed as fold induction from the BM of an uninfected mouse. Lines correspond to means and each dot corresponds to an individual mouse, and data were combined from 2 independent experiments. Data were analyzed using a 2-tailed unpaired Student's t-test.
